# Endogenous hyperinsulinism: diagnostic and therapeutic difficulties

**DOI:** 10.11604/pamj.2019.33.57.18885

**Published:** 2019-05-27

**Authors:** Esma Leila Gouta, Hichem Jerraya, Wejih Dougaz, Mohamed Ali Chaouech, Ibtissem Bouasker, Ramzi Nouira, Chadly Dziri

**Affiliations:** 1 Surgical Department B, Charles Nicolle Hospital, Faculty of Medicine of Tunis, University of Tunis El Manar, Tunis, Tunisia

**Keywords:** Insulinoma, hypoglycemia, endogenous hyperinsulinism

## Abstract

Endogenous hyperinsulinism is an abnormal clinical condition that involves excessive insulin secretion, related in 55% of cases to insulinoma. Other causes are possible such as islet cell hyperplasia, nesidioblastosis or antibodies to insulin or to the insulin receptor. Differentiation between these diseases may be difficult despite the use of several morphological examinations. We report six patients operated on for endogenous hyperinsulinism from 1^st^ January 2000 to 31^st^ December 2015. Endogenous hyperinsulinism was caused by insulinoma in three cases, endocrine cells hyperplasia in two cases and no pathological lesions were found in the last case. All patients typically presented with adrenergic and neuroglycopenic symptoms with a low blood glucose level concomitant with high insulin and C-peptide levels. Computed tomography showed insulinoma in one case out of two. MRI was carried out four times and succeeded to locate the lesion in the two cases of insulinoma. Endoscopic ultrasound showed one insulinoma and provided false positive findings three times out of four. Intra operative ultrasound succeeded to localize the insulinoma in two cases but was false positive in two cases. Procedures were one duodenopancreatectomy, two left splenopancreatectomy and two enucleations. For the sixth case, no lesion was radiologically objectified. Hence, a left blind pancreatectomy was practised but the pathological examination showed normal pancreatic tissue. Our work showed that even if morphological examinations are suggestive of insulinoma, other causes of endogenous hyperinsulinism must be considered and therefore invasive explorations should be carried out.

## Introduction

Endogenous hyperinsulinism is an abnormal clinical condition that involves excessive insulin secretion. It is related in 55% of cases to insulinoma which should be first evoked in each case of endogenous hyperinsulinism [1]. Other causes are possible such as islet cell hyperplasia, nesidioblastosis or antibodies to insulin or to insulin receptor [2]. Differentiation between these different etiologies may be difficult especially in cases where morphological examinations are negative [3]. We report here six patients operated on for endogenous hyperinsulinism while underlining diagnostic and therapeutic difficulties.

## Methods

It is a retrospective and descriptive study that included six consecutive patients admitted at the Surgical Department B of Charles Nicolle Hospital for endogenous hyperinsulinism, from 1^st^ January 2000 to 31^st^December 2015, and operated on with the most likely diagnosis of insulinoma. Data were presented by their median and range values.

## Results

**Figure 1:**
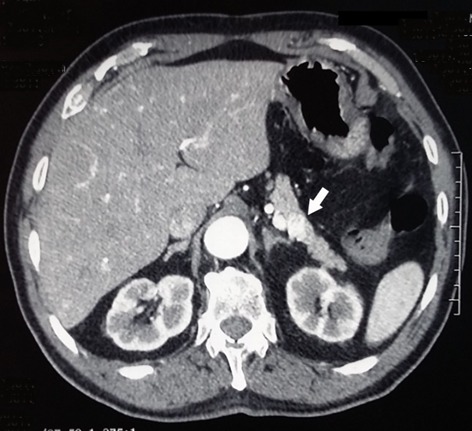
Abdominal CT scan showed a hypervascular lesion of 20mm localized in the tail of the pancreas

**Figure 2:**
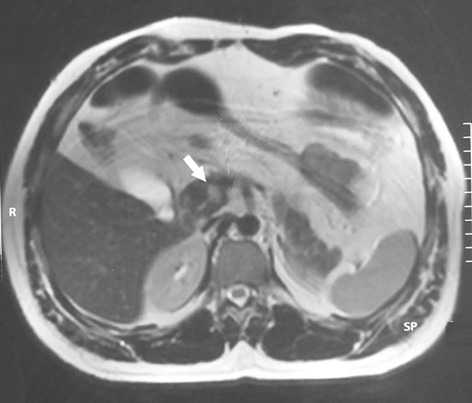
T2-weighted MRI of the abdomen showed a hypervascular lesion of 10mm localized in the head of the pancreas

Six cases of endogenous hyperinsulinism were enrolled over a period of 15 years. All clinical features were summarized in Table 1. Were included four females and two men with a median age of 48.5 years, ranging from 31 to 82 years. Endogenous hyperinsulinism was caused by insulinoma in three cases, endocrine cells hyperplasia in two cases and no pathological lesions were found in the last case. All patients typically presented with adrenergic and neuroglycopenic symptoms. The median time between the first occurrence of symptoms and the diagnosis of endogenous hyperinsulinism was 12 months (range 3-36). All patients had a low blood glucose level concomitant with a high insulin level. The C-peptide level was measured only in five patients. It was high in four cases and normal in one case. Computed tomography (CT) showed insulinoma in one case out of two (Figure 1). Magnetic resonance imaging (MRI) was carried out four times and succeeded to locate the lesion in the two cases of insulinoma (Figure 2). Endoscopic ultrasound (EUS) showed one insulinoma and provided false positive findings in three times out of four. Intra operative ultrasound (IOUS) succeeded to localize the insulinoma in two cases but was false positive in two cases.

**Table 1:** Clinical presentation of the six cases of endogenous hyperinsulinism

Cases	Sex	Age (years)	Symptoms	Duration (months)	Blood glucose (mmol/l)	Insulin (mU/l)	C-peptide (ng/ml)	CT	MRI	EUS	IOUS	Intervention	Pathological examination	Recurrence	Follow up (months)
1	F	44	Palpitation, sweating, headaches, coma	12	0.78	137	1.9	Nl	Nl	++	+	Caudal spleno-pancreatectomy	Islet cell hyperplasia	No	9
2	F	52	Palpitation, sweating	18	4.4	20	*	Nl	*	+	+	Enucleation	Islet cell hyperplasia	Yes (after 1 year)	24
3	M	82	Seizure	3	2.2	32	6.86	+	*	*	*	Caudal spleno-pancreatectomy	Malignant Insulinoma	No	84
4	F	59	Memory loss, fatigue, coma	36	2.29	29	4.33	*	+	++	+	Enucleation	Benign Insulinoma	No	14
5	M	31	Coma	12	-	-	-	Nl	+	*	+	Duodeno-pancreatectomy	Benign insulinoma	No	6
6	F	43	Palpitation, coma	10	2.19	59.2	4.6	Nl	Nl	Nl	Nl	Left pancreatectomy	Normal	No	4

(+/++) number of solid lesion suggestive of insulinoma; (Nl) normal; (*) Not done

Regarding the localization of the detected lesions, procedures were: one duodenopancreatectomy, two left splenopancreatectomy and two enucleations. For the sixth case, no lesions were objectified neither by preoperative imaging nor by IOUS. Hence, a left blind pancreatectomy was practised. However, histological examination was normal. Symptoms of hypoglycemia disappeared for all patients postoperatively. Pancreatic fistula was observed in two cases which dried spontaneously. In five cases out of six, no clinical signs of recurrence were observed after a median follow up of 12 months (range 3-24). In case n°2 (Table 1) corresponding to islet cell hyperplasia, symptoms of endogenous hyperinsulinism recurred one year after and were biologically confirmed. However, imaging explorations (ultrasound (US), CT, MRI and EUS) were negative. A distal pancreatic resection was performed. The postoperative course was uneventful with normalization of blood glucose level. Nevertheless, pathological examination revealed normal pancreatic tissue. She was lost to follow up after 24 months.

## Discussion

Our work showed that, firstly, endogenous hyperinsulinism was associated only in three cases to an insulinoma out of six and secondly, the other causes of endogenous hyperinsulinism must be considered even if morphological examinations are suggestive of insulinoma.

Insulinoma is the most frequent cause of endogenous hyperinsulinism since it is found in 55% of cases in adults [1]. Its incidence is about 1-4 per million [2]. The aim of preoperative imaging is to localize the insulinoma. However, preoperative topographic diagnosis of insulinoma remains a difficult challenge because of low sensitivity concerning conventional imaging techniques for pancreatic small lesions. Indeed 80% of insulinoma have less than 2cm in diameter and 40% less than 1cm [3]. This explains the reported sensitivities of CT and MRI which are respectively about 33%-64% and 40%-90% [4, 5]. In our work, MRI was the most efficient method to detect insulinoma whereas EUS revealed false positive findings three times out of four. EUS can yield misleading findings and its sensitivity to detect insulinoma is about 80% [3]. Mabrut reported in his study a case with a false positive diagnosis of insulinoma related to postoperative fibrosis [3]. Kann also showed that pancreatic nodules of unknown dignity were localized in 1% of 438 cases obtained by EUS [6].

When insulinoma is clinically and biologically evoked, some authors believe that negative imaging explorations do not affect the indication of surgical exploration associated with IOUS [3]. In our study, IOUS succeeded to localize the insulinoma in two cases but was false positive in two cases. De Santibañes revealed that IOUS allowed the detection of the pancreatic tumor from 92% to 94.7% of cases [7]. However, according to Mabrut, the sensitivity of IOUS was 86.3% and the false positive cases were related to postoperative scar fibrosis [3].

Other authors used aggressive strategies to localize the insulinoma in case of negative investigations. Stimulation of insulin secretion in response to calcium injection into a pancreatic artery called Arterial Stimulation Venous Sampling, helps to detect the pancreatic tumor in 90% of cases [8]. It had a sensitivity of 87.5% [9]. Moreover, scintigraphy GLP -1R agonists were recently developed to optimize both pre-operative and intraoperative location of insulinoma with promising results [10]. When insulinoma is not localized, particularly intraoperatively, some authors argue that the procedure should be stopped and the patient referred to a centre capable to perform advanced preoperative and intraoperative localization techniques mentioned above [10, 11]. This would avoid blind distal pancreatectomy for occult insulinoma. In our study, we performed one blind distal pancreatectomy, but the pathological examination was normal. Nikfarjam reported three failed blind distal pancreatic resections for insulinoma. Later on, the three patients were found to have tumors within the pancreatic head [12].

Furthermore, our study suggests that the other causes of endogenous hyperinsulinism should be considered if the results of preoperative morphological imaging are negative or discordant. Endocrine cells hyperplasia is defined by an expansion of the endocrine cell mass to more than 2% in adults [13]. Histologically, all types of islet cells are normally distributed throughout the islets but the predominant cell type is the β cell [13]. Beta cells hyperplasia can only be controlled by partial pancreatectomy, which was done for one of our two patients with a successful result. The second case who had enucleation presented a recurrence of hypoglycemia one year after surgery. The other cause of endogenous hyperinsulinism is nesidioblastosis which represents 0.5 to 7% of all adults [14]. It is revealed clinically by neuroglycopenic and catecholamine response symptoms during fasting. In contrast to patients with insulinoma, such symptoms may occur post prandially [14]. Documented hyperinsulinemic hypoglycemia with a clinically and chemically negative fast test is highly suggestive of nesidioblastosis [15]. Surgical treatment with pancreatectomy is the recommended therapeutic method [2]. Finally, endogenous hyperinsulinism may also be caused by a high titre of antibodies to insulin or by insulin receptor mutations. The autoimmune syndrome is revealed by initial hyperglycemia followed by hypoglycemia a few hours later and biologically with high insulin levels, usually above 100 mU/L [16]. Insulin receptor mutations are revealed by increased fasting levels of serum insulin with normal C-peptide levels. Therapeutic management of both hyperinsulinism caused by autoimmune syndrome or by insulin receptor mutations is not well defined [17].

## Conclusion

Endogenous hyperinsulinism is an abnormal clinical condition that involves excessive insulin secretion, related in 55% of cases to insulinoma. Other causes are possible such as islet cell hyperplasia, nesidioblastosis or antibodies to insulin or to the insulin receptor. Differentiation between different etiologies of hyperinsulinism may be difficult especially when morphological examinations are negative. Our work showed that even if morphological examinations are suggestive of insulinoma, other causes of endogenous hyperinsulinism must be considered and therefore invasive explorations should be carried out.

### What is known about this topic

- Endogenous hyperinsulinism is an abnormal clinical condition that is related to insulinoma in 55% of cases;
- Endogenous hyperinsulinism can also be related to islet cell hyperplasia, nesidioblastosis or antibodies to insulin or to the insulin receptor.

### What this study adds

- Other causes of endogenous hyperinsulinism must be considered and researched whenever the diagnosis of insulinoma is not certain;
- Invasive explorations should be carried out when the aetiology of endogenous hyperinsulinism is not clear enough.

## Competing interests

The authors declare no competing interests.
